# Supplementary material for: Distinct Neutralising and Complement-Fixing Antibody Responses Can Be Induced to the Same Antigen in Haemodialysis Patients After Immunisation with Different Vaccine Platforms
Source: Vaccines (Basel). 2024 Dec 25;13(1):7. doi: 10.3390/vaccines13010007 (PMC11768972; doi:10.3390/vaccines13010007)
Supplement: Supplementary file 1 [file vaccines-13-00007-s001.zip › vaccines-3335608-supplementary.pdf]

Supplementary Table S1. Predictors of anti-S antibody levels in HD patients after 2 vaccine doses. Univariable and multivariable linear regression analysis shown. Multivariable model included all parameters shown. Dependent variable (anti-S antibody levels) was log_{10} transformed for Normality. P values <0.05 considered significant and highlighted in bold typeface.

	**Univariable analysis**		**Multivariable analysis**	
	Beta coefficient (95% CI)	p value	Beta coefficient (95% CI)	p value
Age (decades)	1.00 (0.98, 1.02)	0.84	1.00 (0.98, 1.03)	0.64
Female gender	0.71 (1.35, 2.75)	0.29	0.87 (0.50, 1.53)	0.64
White ethnicity	0.33 (0.19, 0.62)	**<0.001**	0.58 (0.32, 1.04)	0.07
Diabetes mellitus	1.29 (0.66, 2.45)	0.46	0.89 (0.50, 1.60)	0.70
Immunosuppression	0.23 (0.08, 0.69)	**0.009**	0.22 (0.09, 0.59)	**0.003**
Previous SARS-CoV-2 infection	4.07 (2.19, 7.59)	**<0.001**	4.61 (2.60, 8.17)	**<0.001**
mRNA vaccine	5.24 (2.82, 9.77)	**<0.001**	6.14 (3.48, 10.79)	**<0.001**

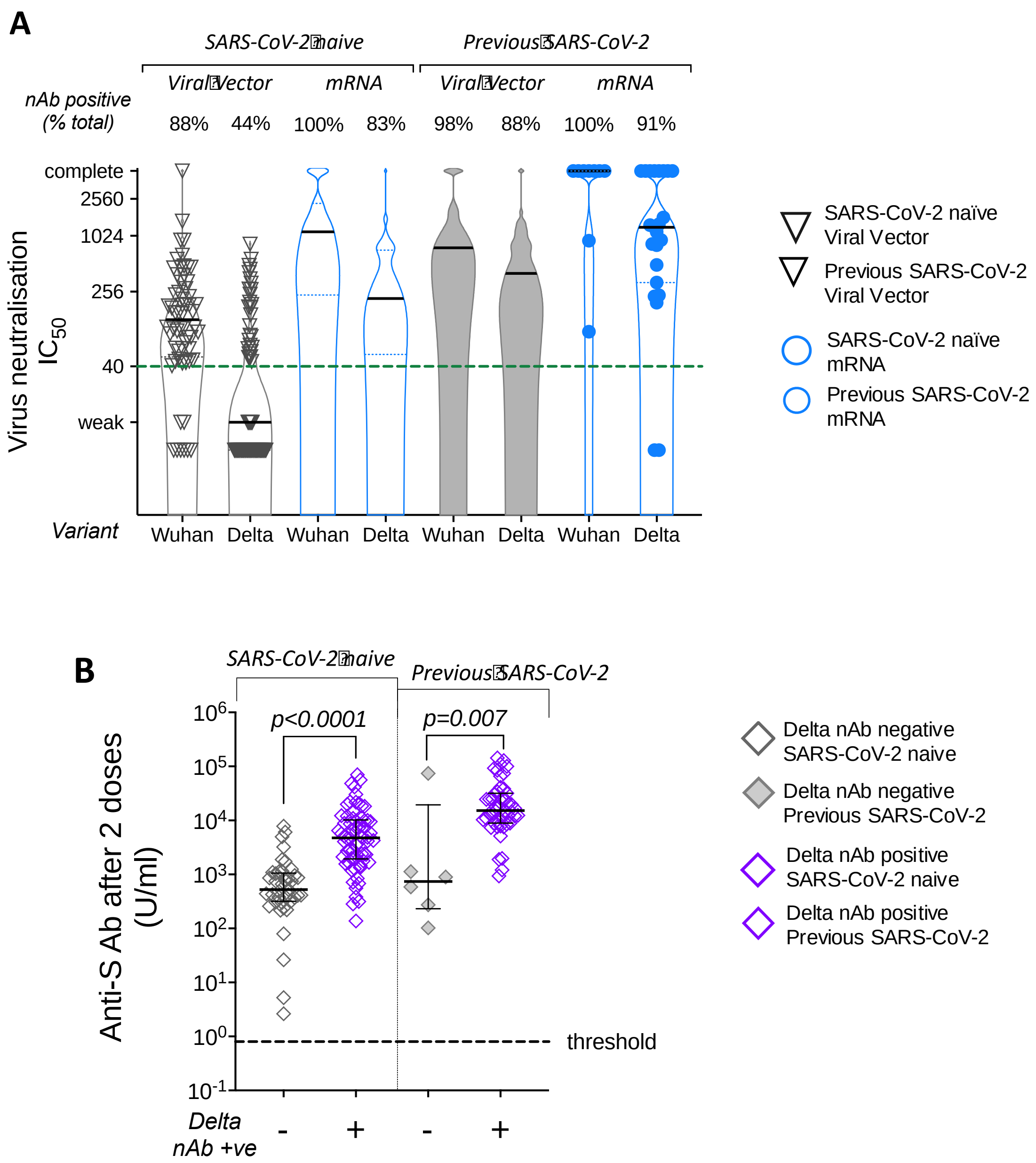

Supplementary Figure S1. Serum virus neutralization activity in HD patients 21-28 days after 2 vaccine doses.
A: Virus neutralisation activity of sera against Wuhan and Delta strains – data split between viral vector (grey symbols) and mRNA (blue symbols) vaccinees and groups split by previous SARS-CoV-2 infection (filled symbols denote previous infection, unfilled symbols – no infection). IC50 of 40 was used as threshold of activity (dashed green line). Percentages indicate proportions of individuals with detectable neutralization activity in each subgroup. **B:** Comparison of anti-S antibody levels between Delta nAb positive (filled symbols) and negative (unfilled symbols) individuals, split by previous infection status. Mann Whitney U test p values shown.

Supplementary Table S2. Predictors of post-vaccination SARS-CoV-2 infection in HD patients after 2 vaccine doses.

Univariable and multivariable logistic regression analysis shown. Dependent variable was incidence of SARS-CoV-2 infection after 2 doses of vaccine (before administration of third dose). Multivariable model included age, gender, ethnicity, diabetes, immunosuppression, HD centre and vaccine type (statistics shown for model with highly functional antibody – Delta nAb +ve with high complement binding). Previous SARS-CoV-2 exposure was significantly co-linear with all measures of antibody functionality and was, therefore, not included in the model.

*Measures of antibody functionality (Delta nAb, complement binding and combined nAb and complement binding) were added individually to the model as they demonstrated significant co-linearity. P values <0.05 considered significant and highlighted in bold typeface.

Abbreviations: OR - Odds ratio; HD – haemodialysis, nAb – neutralizing antibody.

	**Univariable analysis**		**Multivariable analysis**	
	OR (95% CI)	p value	OR (95% CI)	p value
Age (decades)	1.07 (0.72, 1.59)	0.73	1.13 (0.68, 1.87)	0.65
Female gender	0.75 (0.27, 2.11)	0.59	0.47 (0.14, 1.60)	0.23
White ethnicity	0.90 (0.34, 2.40)	0.84	0.37 (0.11, 1.29)	0.12
Diabetes mellitus	0.70 (0.25, 1.96)	0.50	0.36 (0.10, 1.28)	0.11
Immunosuppression	0.56 (0.07, 4.48)	0.58	0.28 (0.03, 2.89)	0.29
HD centre	0.97 (0.36, 2.58)	0.95	1.79 (0.29, 11.1)	0.53
mRNA vaccine	1.06 (0.39, 2.88)	0.91	1.11 (0.17, 7.04)	0.92
Detectable C1q-C5b binding*	0.32 (0.11, 0.88)	**0.028**	0.21 (0.06, 0.70)	**0.01**
Delta nAb +ve*	0.27 (0.10, 0.77)	**0.014**	0.15 (0.04, 0.52)	**0.003**
Delta nAb +ve with high complement binding*	0.49 (0.27, 0.87)	**0.015**	0.34 (0.17, 0.70)	**0.003**

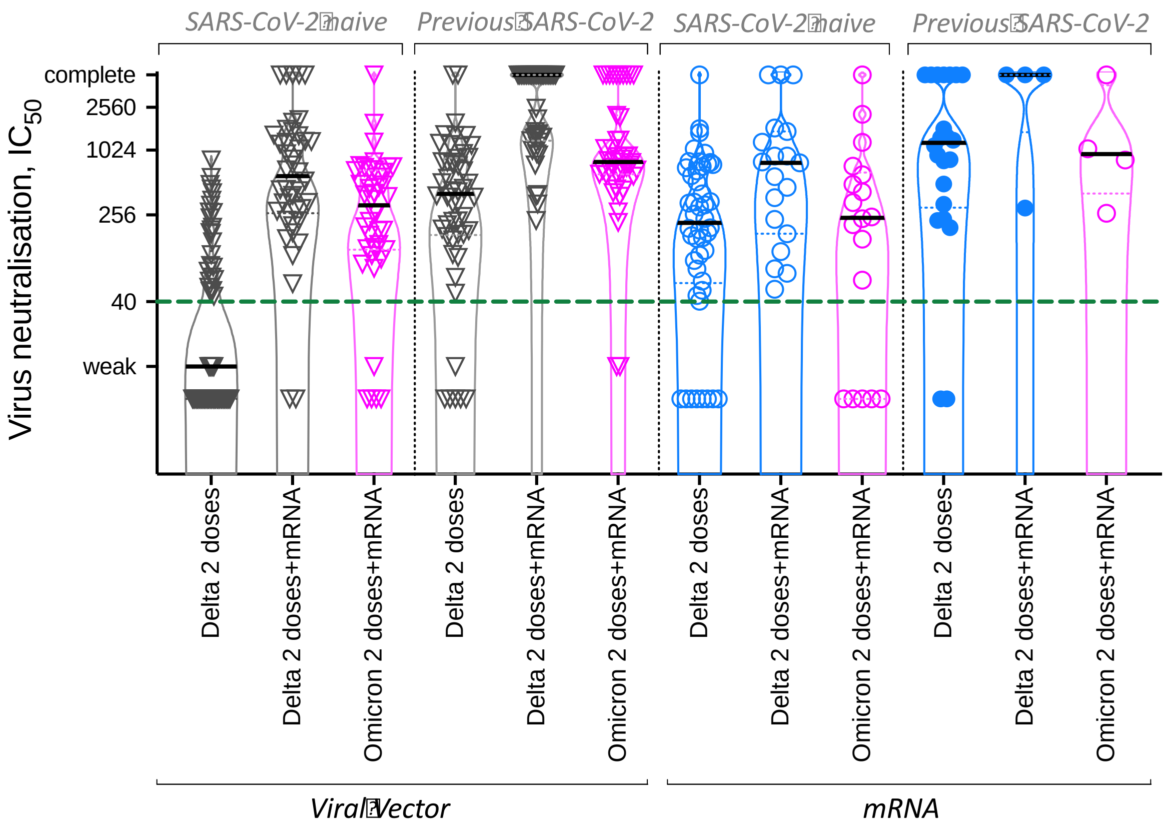

Supplementary Figure S2. Serum virus neutralization activity in HD patients 21-28 days after 2 and 3 vaccine doses.

Virus neutralisation activity of sera against Delta and Omicron variant strains – data split between viral vector (triangle symbols) and mRNA (circle symbols) vaccinees and groups split by previous SARS-CoV-2 infection (filled symbols denote previous infection, unfilled symbols – no infection). IC50 of 40 was used as threshold of activity (dashed green line).

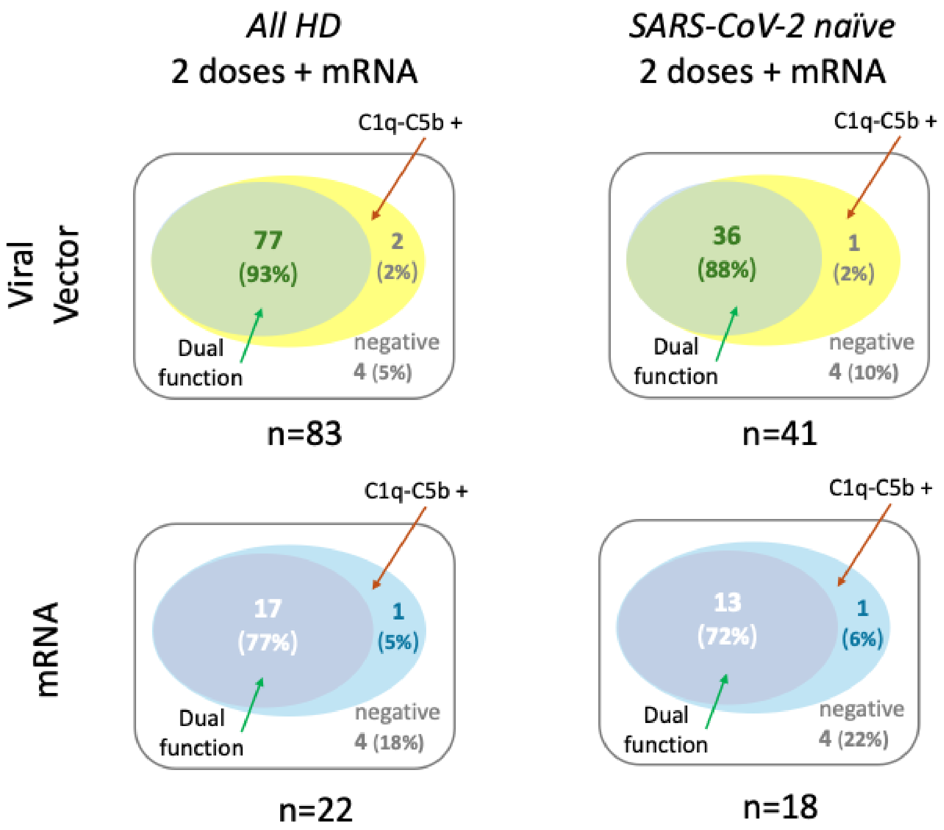

Supplementary Figure S3. Antigen-specific antibody functionality in patients requiring HD after 3 vaccine doses – neutralization activity against Omicron variant.
Diagrammatic representation of antigen-specific antibody functionality after 3 vaccine doses for all HD patients and SARS-CoV-3 naïve individuals. Venn diagrams showing overlap of neutralization activity against Omicron VoC with binding of all 4 complement components tested (C1q-C5b +) to denote antibody with dual function; n and % of total for whom data was available are shown.
